# Motion based prediction and development of response to an "on the way" stimulus

**DOI:** 10.1186/1471-2202-14-S1-P314

**Published:** 2013-07-08

**Authors:** Mina A Khoei, Giacomo Benvenuti, Frédéric Chavane, Laurent U Perrinet

**Affiliations:** 1 Institut de Neurosciences de la Timone, UMR 7289, CNRS, Marseille, France; 2 Aix-Marseille Université, Marseille, France

## 

Prior information on regularities of the world is essential for optimal performance of sensory processing. In the particular case of the detection of visual motion, the prior knowledge on the temporal coherency of motion facilitates estimation of predictable trajectories. Based on such prior information, we have proposed a generic bayesian modeling framework to implement anisotropic diffusion of estimated motion information. By applying particle filtering (Sequential Monte Carlo) we were able to study the emergence of a global neural response through a probabilistic pool of local sensory information. To explore if such diffusive mechanisms could explain neurophysiological recordings from the responses of neural populations to a predictable moving stimulus that we have recorded from V1 neurons in macaque monkeys, we simulated such response in the model. Our main experimental protocol consisted of a bar moving to a fixed direction but with different trajectory lengths before reaching the receptive fields of the recorded neurons. Experimental results show that a bar moving towards a long trajectory generates an anticipatory response that build-up gradually with time before the bar actually enters into the receptive field of the cell. In the modeling part we aim to asses the role of prediction in the dynamical development of neural activity at, or *before*, the arrival of the sensory stimulus.

We simulated the response of the model in target receptive fields to a smoothly moving stimulus whose trajectory crosses the receptive field. This was done under various conditions for two classic stimuli: a dot and a slanted bar. We found that, as expected, a smoothly moving stimulus triggers a transient response as it crosses the receptive field and that he peak time of this response matches the arrival of the stimulus to the it's center. We found that the development of a response to an 'on the way stimulus' is enhanced by increasing the reliability of sensory information along the trajectory of motion. Also we observed an advance of the peak time with respect to the degree of predictability of the trajectory. This is in agreement with the neural activity that we have recorded from V1 neurons in response to a smoothly moving slanted bar with different trajectory lengths. This results may be explained with the definition of classical and extra classical receptive fields while former is activated only by having a stimulus inside it and later covers bigger area and collects information from a wider neighborhood [1]. Motion based prediction by highlighting the anisotropic diffusion of estimated motion information can develop another perspective on lateral spread of activity in primary visual cortex and better understanding of modulatory effect of cells on each other.
